# Assembling the evidence jigsaw: insights from a systematic review of UK studies of individual-focused return to work initiatives for disabled and long-term ill people

**DOI:** 10.1186/1471-2458-11-170

**Published:** 2011-03-21

**Authors:** Stephen Clayton, Clare Bambra, Rachael Gosling, Sue Povall, Kate Misso, Margaret Whitehead

**Affiliations:** 1 Division of Public Health, University of Liverpool, UK; 2 Wolfson Research Institute, Durham University, UK; 3 Public Health Directorate, NHS Sefton, UK; 4 Kleijnen Systematic Reviews Ltd., UK

## Abstract

**Background:**

Employment rates of long-term ill and disabled people in the UK are low and 2.63 million are on disability-related state benefits. Since the mid-1990 s, UK governments have experimented with a range of active labour market policies aimed to move disabled people off benefits and into work to reduce the risk of poverty and social exclusion. This systematic review asks what employment impact have these interventions had and how might they work better?

**Methods:**

A systematic review of observational and qualitative empirical studies and systematic reviews published between 2002 and mid-2008 reporting employment effects and/or process evaluations of national UK government interventions focused on helping long-term sick or disabled people (aged 16-64) into the open labour market. This built on our previous systematic review which covered the years 1970 to 2001.

**Results:**

Searches identified 42 studies, 31 of which evaluated initiatives with an individual focus (improving an individual's employability or providing financial support in returning to work) while 11 evaluated initiatives with an environmental focus (directed at the employment environment or changing the behaviour of employers). This paper synthesises evidence from the 31 studies with an individual focus. The use of personal advisors and individual case management in these schemes helped some participants back to work. Qualitative studies, however, revealed that time pressures and job outcome targets influenced advisors to select 'easier-to-place' claimants into programmes and also inhibited the development of mutual trust, which was needed for individual case management to work effectively. Financial incentives can help with lasting transitions into work, but the incentives were often set too low or were too short-term to have an effect. Many of the studies suffered from selection bias into these programmes of more work-ready claimants. Even though these were national programmes, they had very low awareness and take-up rates, making it unlikely that a population-level impact would be achieved even if effective for participants.

**Conclusions:**

The evidence reveals barriers and facilitators for the effective implementation of these types of interventions that could inform the continuing welfare reforms. The evidence points towards the need for more long-term, sustained and staged support for those furthest from the labour market.

## Background

Recent decades have witnessed an unprecedented expansion in the number of people with chronic (long-term) illnesses and disabilities being outside the labour market across many Organisation Economic Cooperation and Development (OECD) countries [1]. In the UK employment rates of long-term sick and disabled people are low (49%) and 2.63 million are on incapacity-related benefits [2]. This represents around 7.2% of the working age population and the largest group of working age benefit claimants [3].

Since the mid-1990 s various reforms have been introduced with the twin aims of increasing the employment rates of disabled people and of reducing the caseloads and costs of incapacity-related benefits. In 1995, Invalidity Benefit was replaced by the less generous Incapacity Benefit, which included a more stringent eligibility test, with the aim of reducing the numbers of people flowing on to benefits that did not require them to be actively looking for work. The replacement of existing and ineffective employment quotas for disabled people with the rights-based Disability Discrimination Act 1995 was intended to provide a legal barrier to discrimination in recruitment and employment as well as a spur to employers to make work more 'disability friendly.' With the advent of the Labour government in 1997, the UK began to adopt the type of active labour market policies that have been experimented with across many OECD countries [4-7]. These 'activation' polices (e.g. the *New Deal for Disabled People, Pathways to Work*) aim to provide individualised support to people with health and disability-related barriers to employment and are often packaged with other new or existing initiatives or benefits (e.g. *Access to Work, Permitted Work Rules, Disabled Person's Tax Credit*) which provide further, often financial, support for movement into work. These new policies and reforms largely focused on increasing the individual's 'employability' and 'making work pay' [8]. Continuing reforms saw Incapacity Benefit (IB) replaced in October 2008 by the Employment and Support Allowance. This two-tiered benefit includes a more stringent *Work Capability Assessment *following which those claimants deemed to be capable of work will receive lower benefits than those judged unable to work and receipt of benefit is contingent on them attending work-focused interviews [9].

Whilst many OECD countries have adopted similar activation policies for disabled people, evaluation of such policies and evidence of their effectiveness remains limited. Our own earlier systematic review [10], found only 16 studies reporting evaluations of UK 'welfare-to-work' policies for the long-term sick and disabled from 1970 to 2001. Whilst the review found some evidence of positive employment effects from these programmes, it was difficult to determine how much of this resulted from the specific programmes as only two studies had controls. The studies provided little evidence of any differential impacts by socio-economic or demographic groups. Similarly, a 2008 systematic review for the National Institute for Health and Clinical Excellence (NICE) [11] identified only three experimental studies, none of which provided clear evidence of the effectiveness of the interventions. An international 'best evidence synthesis' of vocational rehabilitation interventions [12] uncovered limited evidence on return to work outcomes, as did a UK synthesis of welfare-to-work studies [10].

Arguably, these existing reviews have not addressed the vital questions of 'why' and 'how' major UK initiatives did or did not work. The answers are not only essential for future policy development, but also for the effective implementation of existing initiatives. To address these questions, insights from both observational and qualitative studies are needed. This article sets out to draw insights from a comprehensive evidence synthesis on the question of 'what helps long-term sick and disabled people into employment in the UK and how might these interventions be made more effective?'

## Methods

### Gathering and synthesizing the evidence

As part of a wider international evidence synthesis [13], we carried out a systematic review of the effectiveness of major UK interventions (i.e. national initiatives instigated by the government) aimed at helping long-term sick or disabled people in the UK into work.

### Inclusion and exclusion criteria

The review aimed to identify all experimental and observational studies, evaluating the employment effects of major interventions in the UK, along with qualitative studies investigating how or why an included intervention was or was not effective. We included measures aimed at helping people age 16-65 with limiting long-term illness or disability into work who were not employed and were on some form of incapacity-related benefit. We excluded studies that were not based on empirical research or that did not include employment in the open labour market as an outcome. We excluded measures aimed at reducing short-term sickness absence among people who were in employment. We only included national initiatives instigated by the government and excluded studies of small-scale, localised interventions. We also excluded evaluations of interventions that focused wholly or largely on sheltered employment as these were rarely concerned with transitions into the open labour market. (See Additional File 1 for full details.)

### Search strategy

As our earlier review [10] covered the years 1970 to 2001, we restricted our search to studies published between 2002 and mid-2008. Sixteen electronic databases were searched by an information specialist using terms developed in consultation with the project team. In addition, we searched the websites of 49 relevant UK governmental, non-governmental, research and charitable/voluntary sector organisations. The bibliographies of all assessed material were hand-searched, and information on unpublished and in-progress research was requested from 26 key researchers in the field. (See Additional File 2 for full details.)

### Critical appraisal

The reviewers excluded clearly irrelevant titles and abstracts and retrieved full text copies of the remainder. All retrieved papers were evaluated for relevance by two reviewers in accordance with the inclusion and exclusion criteria drawn up by the authors. The quality of the studies was assessed using criteria adapted from existing checklists for both quantitative and qualitative studies. The appraisal results were used for descriptive purposes only, to highlight variations in the quality of studies. They were not used to calculate a quality score as this would not be appropriate, given the diverse range and purposes of the studies. Care was taken, however, to consider the design and conduct of each study when interpreting the findings and to be properly cautious in inferring causation. (See Additional File 3 for full details.)

### Synthesis

A total of 42 UK studies were identified that met the inclusion criteria for the systematic review (see Additional File 4). The interventions identified reflect the two principal orientations of UK government-led active labour market policies. One centres on attempts to make the employment environment more 'disability-friendly'. The second aims to increase the 'employability' of the disabled individuals themselves, through developing their skills, education etc., or by providing them with financial support to assist their movement back into work. We classified the interventions by their underpinning programme logic or 'theory of change' depicted in Table 1 which is the explicit or implicit reasoning about how the intervention will bring about the desired change in the perceived problem [14]. This typology was developed as part of a wider international evidence synthesis [13] in order to be able to classify interventions with similar programme logics and then compare the effectiveness of the different approaches and the barriers to implementation.

**Table 1:** Typology of interventions to help long-term sick and disabled people into work

Focus	Intervention type and programme logic	Examples of interventions
**work environment**	**Tackling discrimination** Legislate to outlaw discrimination by employers against disabled/long-term sick in recruitment and retention of staff	*Disability Discrimination Act 1995*
	
	**Improving workplace and employment accessibility** Legal or financial measures to remove or reduce barriers to accessibility of work and employment for the disabled/long-term sick	*Access to Work *scheme
	
	**Offering financial incentives to employers** Job creation or financial incentives to employers to employ disabled and long-term sick to increase employment opportunities.	*Job Introduction Scheme; Work Trial*
	
	**Enhancing return to work planning** Improve provision for planned return to work and for agencies to cooperate and integrate services offered	Forthcoming *Fit for Work *service
	
**individual**	**Offering financial incentives/disincentives for welfare claimants** Financial incentives/reduced benefit generosity to increase incentives to gain employment	Tax credits; *Return to Work Credit, Job Preparation Premium*
	
	**Individualised case management and job search assistance** Individualised vocational advice/job search assistance on a case management basis	*ONE Advisory Service; New Deal for Disabled People; Pathways to Work*
	
	**Education, training and work trial** Improve claimants' skills, education and training to increase 'employability'	*Residential Training; Work Preparation; Work Trial*
	
	**Health condition/impairment management** Medical rehabilitation and/or advice on health condition management to improve fitness to work.	*Condition Management Programme*

Source: Adapted from Whitehead et al, 2009 [13], Table five.one

Of these 42 studies identified, 31 were categorised in the typology in Table 1 as orientated towards the individual and therefore the focus of this paper. (A further paper examines the effectiveness of efforts to make the employment environment more disability friendly). These 31 studies evaluated three national programmes piloted or established since 1997: the *ONE Advisory Service*, the *New Deal for Disabled People *and the *Pathways to Work *programme and other initiatives packaged with them (*Return to Work Credit, Permitted Work Rules, Condition Management Programme*). Implementation details for all these initiatives can be found in Additional File 5. Study designs ranged from large-scale controlled cohorts to small, qualitative studies of staff and clients. Two reviewers independently read and re-read the individual papers and tabulated details of the study design, intervention design and context, participant details, outcomes (e.g. employment, income, health), and factors affecting the operation of the intervention, whether positively or negatively. The two reviewers then discussed their findings to resolve any disagreements. Finally, we synthesised the evidence using a narrative approach [15] in which we report the key employment, health and income impacts using the quantitative studies, and use the qualitative studies to explore the factors that influenced these outcomes in terms of the intervention's context, implementation and target population.

## Results

Studies were identified that evaluated initiatives in three of the 4 categories of individual-focused interventions in the typology in Table 1. There were no evaluations of 'education, training and work-trial' types of intervention, even though interventions of this type have been implemented in the UK over the years. We report the findings of the three identified categories in turn.

### Interventions offering individual case management and job search assistance

Providing individualised support to help people to find work is a strategy adopted throughout Europe. The underlying logic is that people who have been economically inactive for some time may need support in regaining or updating skills in locating and obtaining work. Tailored support (such as access to training, job search advice and in-work benefits advice) provided though Personal Advisors and case management aims to reconnect more claimants to the labour market and also aims to shift the culture of the benefits system from one generating dependence to one promoting activity. UK programmes have also included integration of the employment and benefits services and the provision of services by the private and voluntary sector, on the assumption that this will improve efficiency and encourage innovation [16,17]. A total of 27 studies of this type were found, full details of which are given in Additional File 6. They related to the relevant aspects of the *ONE Advisory Service, New Deal for Disabled People *and *Pathways to Work *pilots, as follows.

#### ONE Advisory Service evaluations

The *ONE *programme integrated the Employment Service and Benefits Agency into a single point of contact tailored to the needs of individuals. New and repeat claimants were assigned a Personal Adviser to process their benefit claim and, through work-focused interviews, review their job readiness, options for work, and barriers to work and provided services such as a better-off calculation and advice about in-work benefits. The service was introduced in 12 pilot areas between 1999 and 2001 using three models: Basic, Call Centre and Private/Voluntary Sector (PVS). From April 2000 sick/disabled claimants were required to attend a work-focused meeting as a condition of receiving benefits in the pilot areas.

Five evaluation studies of this intervention were identified. A controlled cohort study (n = 4783) evaluating the *ONE Advisory Service*, found that employment rates of 16 hours or more per week for sick/disabled clients increased in both the intervention (24% to 28%) and comparison (20% to 25%) areas, but the difference was not statistically significant [16]. The study reported a significant (p < 0.001) increase in employment outcomes for Basic delivery model clients working 16+ and 30+ hours per week, but the authors could find no clear reason why the Basic model should be more effective than the private-voluntary sector or call centre models. A study using a repeated cross-sectional survey of a 5% random sample of UK benefit claimants (n = 29,451), found no statistically significant difference in the probability of sick/disabled clients leaving benefits [18,19]. Additionally, claimants who participated in the early phase of the intervention left benefit quicker than those who participated in later stages. This suggests a worsening of labour market outcomes when the service should have been improving after any start-up difficulties, indicating a possible cohort effect.

Qualitative studies of 103 clients [17] and 72 staff [20] reported that both groups viewed the one-stop-shop approach and the personalised service as significant improvements on previous arrangements. However, both reported that Personal Advisor meetings were heavily focused on sorting out benefit claims, restricting time available for work-related activities, particularly for sick/disabled clients. Clients felt that there was limited advice about or referral to external sources of further support or training. Staff reported that: staff shortages meant that Advisors lacked sufficient time to focus on work-related activity; many Advisors felt ill-equipped to deal with clients with more complex health or personal problems and had a limited knowledge of services to which they could refer such clients; and Advisors were less likely to pursue work-related activities with sick and disabled clients who they largely perceived as not work ready. The introduction of the *Jobcentre Plus Pathfinders *in March 2002 may have created the worsening employment outcomes. The staff study suggests this created insecurity and uncertainty among staff who began leaving the *ONE *pilots. As the pilots had under 12 months left to run, experienced replacement staff were difficult to recruit. Neither study provides useful evidence about the differences in provision between the three models of delivery.

#### New Deal for Disabled People evaluations

Under the *New Deal *(piloted from 1999 and extended nationally in 2001), claimants of incapacity benefit voluntarily undertake work-focused interviews to access an individualised package of job search activities, access to appropriate training and other employment advice (including in-work support for those gaining employment) delivered through a network of private, voluntary or public sector *Job Brokers*.

A total of 12 evaluation studies of this initiative were identified. A large retrospective controlled cohort (n = 522,596 intervention, n = 44,049 control) [21] reported significant (p < 0.05) increases in employment rates after 24 months for both existing (+11%) and new (+7%) claimants. It also found significant (p < 0.001) reductions in benefit recipiency for both existing (-16%) and new (-13%) *New Deal *registered IB claimants. Stronger and significant employment effects were reported for those claiming IB for at least 3 years, those furthest from the labour market, and those in areas with higher rates of IB claims. No substantial differences in employment impact by *Job Broker *type for either new or existing claimants were reported. The study does not adequately adjust for potential selection bias deriving from the voluntary nature of the *New Deal*, with more motivated, job-ready claimants more likely to volunteer [21].

An uncontrolled longitudinal cohort survey of 4082 *New Deal *registrants [22-24] found that after 12 months, 47% of registrants were in paid work. They reported significantly higher likelihood of employment after 12 months for women compared with men (Odds Ratio = 1.24, p < 0.01), for those over 50 (OR = 1.39, p < 0.05), those without basic skills problems (OR = 1.4, p < 0.01) and those whose health condition did not limit their activities (OR = 1.99, p < 0.05). Ethnic minorities (OR 0.73, p < 0.05), those without musculoskeletal (OR 0.50, p < 0.001) or mental health conditions (OR 0.65, p < 0.05) were less likely to be in work. Regional differences were also reported with registrants in London (OR 0.52, p < 0.001), the North West (OR 0.66, p < 0.001), and the West Midlands (OR 0.47, p < 0.001) significantly less likely to enter work. Of those who gained employment, 72% reported that they would have obtained work without the assistance of *Job Brokers*, again suggesting that *New Deal *registrants were already more motivated to return to work.

Qualitative studies of the *New Deal *revealed that participants held mixed views about the role of the intervention in helping them into work. Many emphasised the importance of establishing and maintaining a good relationship with the *Job Broker *adviser, who could be an important source of support, confidence-building and encouragement in moving into or towards work [25,26]. Similarly, qualitative studies with employers and with *Job Brokers *strongly emphasised how the skills of the staff and their ability to establish good relationships (with claimants, Jobcentre Plus and employers) were central to *Job Brokers *success [26-29]. These evaluations did not systematically examine differences between *Job Broker *types. However, one small qualitative study [30], reported that participants' disillusionment with public sector and government, a lack of trust in the latter and fear of benefit withdrawal inhibited claimants' commitment to the programme, particularly when delivered within the public sector.

Many of the studies provided clear evidence of widespread selection into the programme [22-27,29,31,32]. This reflected low awareness of the initiative amongst both the eligible population [31,32] and employers [27,28], and resulted in only around 10% of eligible claimants making contact with *Job Brokers *[31,32]. Claimants often explained their non-participation as due to their health condition being too serious to consider returning to work, although many felt they might register if their condition improved [22-26,30]. Thus selection into the programme partly resulted from individuals' awareness of the programme or feeling that they were suitable candidates. A repeat cross-sectional survey of 3452 IB recipients estimated that only around 7% of existing claimants and 15% of new claimants were 'good candidates' (i.e. those looking for work and willing to use this type of service) for *Job Broker *recruitment [32].

Other qualitative studies [25,26] reported that some *Job Brokers *would not register clients they felt needed higher levels of support than they could provide and others would 'manage' the registration process to weed out clients not 'committed' to the programme. Job conversion targets were viewed as creating pressures on both *Job Brokers *and *Jobcentre Plus *staff to select the more job-ready into the programme [26,29]. That there was some degree of selection of the most job-ready in order to meet targets is supported by data from the survey of registrants which indicated that 14% of registrants entered work within one week of registering and 32% within one month [24].

#### Pathways to Work pilots evaluations

In the seven *Pathways *pilot areas, all new and repeat IB claimants underwent mandatory work-focused interviews eight weeks into their claim, with the possibility of five more at monthly intervals. (*Pathways *was also open to existing claimants in these areas on a voluntary basis, although take up from this group was very low.) Non-attendance could result in benefit deductions. Personal Advisors provided individualised advice and support to facilitate claimants' return to work, including the *Choices *package (easier access to existing programmes such as the *New Deal*, along with new initiatives such as the *Return to Work Credit*, and the *Condition Management Programme*). *Pathways *national roll out was completed in 2008. *Pathways *remains mandatory for new Employment and Support Allowance claimants in the "Work-Related Activity Group" and is available on a voluntary basis for existing incapacity benefits claimants [3].

Ten evaluation studies of Pathways were included in the review. A prospective controlled cohort study of 8035 recipients in 7 pilot areas [33], reported that after 10 1/2 months, the intervention group showed increases in both the probability of being employed (+ 9.4% p < 0.001) and increased monthly earnings (+£71.73, p < 0.001) and reductions in the probability of claiming incapacity benefits (- 8.2%, p < 0.001) and reporting a work-limiting health condition (-2.9%, p < 0.05). No differences were found by age or sex. A follow-up controlled cohort study (n = 5784) [34] reported an increased likelihood of being employed after 18 months (+7.4%, p = 0.09) for the intervention group. This was stronger for women (13%, p < 0.05) and those with dependent children (17.6%, p < 0.05). No statistically significant differences were found for monthly earnings, the probability of claiming incapacity-related benefits or reporting limiting health conditions. As the *Pathways *pilots were only open to new incapacity benefits claimants (less than 10% of the claimant population), both studies were open to potential problems of selection bias that were not adequately accounted for.

As with the *New Deal*, the qualitative studies of *Pathways *indicated that the structured attention provided by Personal Advisors to claimants may have positively affected some claimants' attitudes towards employment. Establishing relationships of mutual respect and trust between Personal Advisors and claimants was widely viewed as the basis for helping the claimants move towards or into employment [35-39]. Claimants' views of the value and purpose of work-focused interviews were mixed [40-42] with only those already close to the labour market indicating that participation had positively affected their views and behaviour towards work [41]. A longitudinal qualitative panel of claimants [40-42] concluded that claimants generally felt that *Pathways *failed to overcome what they perceived as primary barriers to work - weak local labour markets, attitudes of employers - and those with more serious health conditions felt they had nothing to gain from *Pathways*.

Studies with Personal Advisors [36,37,39] and the extension of *Pathways *to existing claimants [35,38] revealed some of the implementation issues and limitations of the existing programme. Advisors tailored their support to claimants based on their proximity to the labour market and key to the process was changing claimants' attitudes towards work. Thus, 'customer progress' for those furthest from the labour market might simply involve getting them to think about moving towards work. The attitudes, skills and workloads of Advisors were reported as key to engaging claimants in the process, although some Advisors were unsure whether 'movement toward work' was a justifiable use of *Pathways *resources. Some Advisors reported prioritising those clients who were likely to return to work quickly, in order to meet targets and be cost effective. Advisors felt that increasing caseloads and the short-term nature of many *Pathways *components limited their ability to provide the long-term engagement needed for those furthest from the labour market, particularly those with more complex (often mental) health conditions. Many Advisors felt that the increasing use of job outcome targets and funding restrictions could undermine their efforts. A further constraint derived from an inherent negative tension between their roles as 'enablers' (providing individualised and supportive interventions) and 'enforcers' (imposing benefit sanctions for non-attendance at work-focused interviews).

### Interventions offering financial incentives for disabled people

Financial incentives have been used both to assist disabled and long-term sick benefit recipients in returning to work, and to maintain their income while in work. The logic of these types of intervention is that people on welfare benefits may fear the loss of income if they take up a part-time or low paid job and so need additional income to smooth the transition from welfare benefits to work. A total of 8 studies of this type of intervention were identified, full details of which are included in Additional File 7.

#### Return to Work Credit and Disabled Person's Tax Credit

The *Return to Work Credit*, introduced as part of *Pathways*, consists of a payment of £40 per week for up to 52 weeks to new IB claimants returning to work for 15+ hours per week and earning less than £15,000 p.a. Six qualitative studies examined the views and experiences of *Return to Work Credit *recipients [40-44] and staff administering the *Credit *[36,39].

All these studies indicated that the *Credit *can assist with lasting transitions from benefits to work through helping with day-to-day budgeting, clearing existing debts and boosting people's confidence about their financial situation once in work. Some recipients reported, however, that this extra support had been eroded by factors such as the activation of debt recovery [43]. Staff felt that the *Credit*'s incentive effect only worked for those already close to the labour market and some were concerned that it only provided support for low paid work [36,39].

A longitudinal qualitative panel survey of 106 incapacity benefit claimants [40-42] reported limited uptake of the *Credit*, with the highest uptake among women returning to low-skilled, part-time work. Overall, the studies concluded that whilst the *Credit *may have provided an incentive or support for claimants already thinking about returning to work, there was no clear evidence that their return to work depended on it. Similar findings were reported for the *Disabled Person's Tax Credit*, with recipients reporting that it provided extra financial security which helped movement into work, but overall take up was low [44].

#### Permitted Work

Originally introduced in 2002 to replace *Therapeutic Work*, the *Permitted Work *rules allow incapacity benefit claimants to work up to 16 hours per week and earn up to £88.50 per week for up to 52 weeks (or earn £20 per week indefinitely) without losing benefits. An uncontrolled (and unrepresentative) cohort survey of 1435 *Permitted Work *claimants [45,46] found that at the final interview 58% of respondents were in work, 33% of whom were still claiming incapacity benefit under *Permitted Work*. Under the *Permitted Work *scheme, individuals (particularly those with a shorter benefits history or with a working partner) were more likely to start work than under the previous *Therapeutic Work *scheme. The authors noted that single people faced a benefit trap as the threshold earnings cut off for *Working Tax Credit *was higher for couples, which made working over 16 hours more viable for them [46].

### Management of health conditions to improve fitness to work

Long-term sick and disabled people can potentially be helped back into work though assisting them to manage their particular health condition better in order to reduce its work limiting effect. The *Condition Management Programme (CM Programme)*, introduced as part of *Pathways*, is one of the few UK examples of this type of intervention. It was designed to address the three main conditions reported by those claiming incapacity benefit - mental health issues, cardio-vascular and musculoskeletal problems. Commissioned and delivered jointly by Jobcentre Plus and Primary Care Trusts, the *CM Programme *attempts to tackle deep-seated issues such as anxiety, pain management and lack of confidence, with programme delivery and content varying according to local needs. A total of seven studies evaluating this type of intervention were reviewed, full details of which are in Additional File 8.

A common theme across the seven qualitative studies was that the *CM Programme *assisted claimants to move towards, if not actually into, work. A qualitative case study which matched existing incapacity benefit claimants and Personal Advisors [38], reported that Advisors viewed the *CM Programme *as the most appropriate referral option for those claimants furthest from the labour market as it provided them with the necessary first step of exploring how they might deal with their health conditions on a day-to-day basis. Another theme was how, in the *CM Programme*'s pilot phase, Advisors often lacked a clear understanding of the scheme and had insufficient knowledge of health conditions, particularly mental health conditions, which could lead to inappropriate referrals and unsuccessful outcomes. A longitudinal panel survey of 106 incapacity benefit claimants [40-42] reported positive views of the *CM Programme *in terms of managing and improving health conditions, but, of those who had returned to work, only some accredited this to the programme. There was also some indication that focusing on specific conditions was more effective in moving clients towards work and improving health conditions than generic approaches. A study of the views and experiences of *CM Programme *staff [47] emphasised that staff considered that successful outcomes resulted from having motivated clients undertaking the *CM Programme *alongside other *Jobcentre Plus *interventions. Staff generally felt that those clients who were not progressing had more complex personal problems that required further specialist help.

## Discussion

### Insights for future programmes

The review found evidence on the impact and implementation of various strategies with an individual orientation within three national UK policy programmes: the *ONE Advisory Service, New Deal for Disabled People *and *Pathways to Work*. What lessons are there for planning future policies and improving existing initiatives in this area?

One key finding for future programmes is the need for more effective and long-term evaluation of any programmes initiated by central government. No randomised controlled trials (RCTs) were identified in our searches, and whilst those observational studies using comparison groups or areas provided reasonably robust evidence, most did not adequately control for or discuss the problems of selection bias in both the intervention and comparison groups. This limits the conclusions that can be drawn about the employment effects reported in these studies. Overall the qualitative studies were well-designed although, given the space available in these extensive reports, relatively few provided adequate details of how the findings and conclusions were derived from the data. The multi-method comprehensive programmes of evaluation, such as those for the *New Deal *and *Pathways *provide good models of how to evaluate both the effects and the processes of complex social policy interventions. One notable absence, however, is that of well-designed longitudinal studies. Such studies may address a number of questions raised by the studies reported here, including whether the employment outcomes reported were sustainable

There was evidence from several studies that personal advisors and individual case management in these schemes helped some participants back to work, but there was widespread selection into these programmes of more work-ready claimants, which biased the results of the evaluations. This also resulted in inequalities in access to the support on offer. Claimants 'job readiness', age, type of disability or health condition or family situation, as well as local labour market conditions and welfare benefits context influenced whether an incapacity benefit claimant was selected or not. This included the self-selection deriving from the voluntary nature of work-focused interviews under *New Deal *and from the fact that whilst these were mandatory under the *Pathways to Work *pilots, they were only open to new claimants.

In addition, Personal Advisors across the initiatives were more willing to work with claimants who were more work-ready, often in order to fulfil job outcome targets or other organisational needs. From an evaluation perspective, selection into programmes of those claimants closer to the labour market makes it difficult to assess how effective these programmes can be with those more distant from the labour market, and how programmes might be changed to increase their effectiveness for these groups.

The problem of selection also hindered the interpretation of evidence of differential impact. There was an intriguing finding of differential impact from one *New Deal *study, for example, with a greater likelihood of employment following participation in the programme for participants who had been claiming incapacity benefit for three years or more, those furthest from the labour market and those living in areas with the highest rates of economic inactivity. The voluntary nature of *New Deal *and the selection activities of advisors, however, coloured the interpretation of these findings so that we cannot draw conclusions about whether the differential effect was robust. Examining the employment impact of *Pathways*, one report [33] found no significant difference by gender, whereas the follow up [34] found a larger positive effect for women and for those with dependent children. Neither the quantitative nor qualitative studies of the *Pathways *pilots provide any clear explanation of what aspect of the programme might be producing these differential effects.

A recent assessment of the effectiveness of *Pathways to Work *[3] noted that the positive employment outcomes found in the pilot studies have not been replicated since the programme was rolled out nationally. While new claimants moved off benefits sooner than without the programme, around 80% of this was due to claimants failing the medical assessment at an earlier stage in *Pathways *areas rather than their participation in the programme. The assessment also notes that recent evaluations indicate that once on incapacity-related benefits, new claimants are just as likely to move into employment without *Pathways *support as with it. The report concluded that the obligatory work focused interviews and early medical assessment in *Pathways *were the key aspects of the programme that move people off benefits and into work, whilst the voluntary components, such as the *Condition Management Programme *and *Return to Work Credit*, apparently had no additional employment impact. The assessment, however, did not provide data on differential effects, nor did it take into account the problems of selection identified in our review. As *Pathways *has largely been applied to new and repeat claimants, who are much more likely to return to the labour market unaided, it is perhaps not surprising that its voluntary components show no additional employment effect.

Another issue for these programmes, somewhat mitigated in *Pathways to Work *by its mandatory nature, was that awareness of the schemes and reach among the target population of incapacity benefit claimants was generally low. The overall take-up rate for the *New Deal for Disabled People*, for example, amounted to just over 3% of the eligible population between 2001 and 2006 [48]. Uptake of return to work and in-work benefits was also low. From a policy perspective, it is important to build into national programmes improved arrangements for promoting awareness of the services and incentives on offer, together with careful monitoring of differential access and outcome of initiatives.

The studies reviewed were mostly conducted between 2000 and 2006, a period of general employment expansion in the UK, but this was not associated with a reduction in the stock of incapacity benefit claimants [49]. Whether the positive (if limited) employment outcomes indicated for some programmes would be maintained in less favourable employment conditions remains to be tested.

Each of the three main programmes under review here employed work-focused interviews as the main means of assessing claimants' work-readiness and designing an individualised package of assistance aimed at assisting their movement into the labour market. The qualitative studies provided valuable insights into the difficulties of implementing and operating these individualised active labour market programmes. A common theme in the qualitative studies with Personal Advisors was that helping those furthest from the labour market (i.e. the majority of existing claimants) requires more time than is often acknowledged by programme managers or within the job outcome targets. Linked to this is that the achievement of positive outcomes relied on the building of trust through the creation of understanding, positive and supportive relationships between the staff and claimants, usually on a one-to-one basis. Personal Advisors and claimants saw potential barriers to the establishment of such trusting relationships in the limited time available for personal contact, in claimants concerns about potential income reduction through loss of benefits and in that part of the personal advisor role which enforced benefit sanctions for failure to attend work-focused interviews. Finally, the targets that advisors were set were focussed largely on how many disabled people were moved off benefits and into work, and ignored important improvements in moving people closer to getting a job. There was some suggestion that such targets encouraged 'cream-skimming', particularly among private providers of Job Broker services.

The studies reviewed provide little evidence on the comparative effectiveness of public, private and voluntary sector delivery models. The *ONE *studies reported no differences in employment outcomes between the Private and Voluntary Sector (PVS) model and the other models. The poor performance of the PVS model resulted in it being omitted from the subsequent *Jobcentre Plus Pathfinders *programme [50]. Furthermore, the 2010 National Audit Office assessment of *Pathways to Work *concluded that Jobcentre Plus *Pathways *had performed better than the voluntary/private sector provider-led *Pathways *in terms of getting mandatory participants into work [3]. Despite this evidence, proposed reforms in the UK still favour the private and voluntary sector model on the (unsupported) assumption that it is more effective.

The findings suggest that the financial incentives for disabled people built into the national programmes, such as the *Return to Work Credit *and *Permitted Work*, can support the process of transition into work. They also highlighted obstacles to the effectiveness of the schemes. The financial incentives were set too low or did not last long enough to assist with transitions into employment, particularly where this meant that other benefits (e.g. housing benefit, council tax benefit) were reduced when returning to work. These incentives also had low take up due to low levels of awareness among claimants or restricted entitlement to the incentives (the latter is particularly the case with *Permitted Work*).

A distinct shift in thinking in the UK on what strategies might help disabled people into work was signalled with the introduction the Condition Management Programme as part of *Pathways to Work*. Whilst the studies reviewed here did not provide evidence of the programme's employment impact, they provided useful insights for policy. The studies generally supported the themes noted in the Personal Advisor studies, that reintegrating claimants furthest from the labour market requires longer-term and personal engagement with individuals. This seems to be particularly critical in relation to people with mental health conditions and the intensive support needed to help them into work [51].

## Conclusion

### Implications for recent reforms

Reforms to the unemployment benefit system in general, not limited to benefits for people with disabilities, were introduced in the UK in 2008. These were based on vision of a personalised service suitable for all unemployed groups, coupled with strict conditionality attached to the receipt of the benefits [52]. As part of these reforms, the *Employment and Support Allowance *(ESA) replaced incapacity benefit for new claimants in October 2008. Claimants undergo a more stringent assessment, the *Work Capability Assessment*, and if assessed as able to undertake work related activity receive a lower level of benefit than those judged unable to work because of sickness, conditional upon their participation in return to work activities [9]. The *Work Capability Assessment *was extended to all existing claimants of incapacity benefits from early 2011. Department for Work and Pensions statistics show that of the 496,200 assessments up to the end of May 2010, 66% of applicants had been deemed fit for work. Around 32% of claimants appealed this decision, however, and about 40% had their appeals upheld [53].

The evaluations of the previous programmes point to a number of potential implications for this new scheme. The pressures on Advisors (whether in the public, private or voluntary sectors) to select more work-ready individuals into programmes will remain if job placement targets remain the primary measure of programme effectiveness. Incorporating some measure of 'movement toward work' as part of the judgement of effectiveness may lessen the pressures to select the most work-ready. If those claimants who are being rejected by the new assessment process remain out of work, they will lose access to services which might have eased their movement into work, a generic problem noted in one study in our review [42]. As the qualitative studies reveal, claimants' motivations to engage with programmes can be undermined if they feel the purpose is just to move them off disability benefits. The Advisor studies emphasise the need for more long-term, sustained and staged support to help those furthest from the labour market. Other studies [54,55] have suggested this requires a flexible package of long-term support that acknowledges that for some claimants work is not an immediate option. One example of such a flexible package that has been suggested in a Department for Work and Pensions policy review [51] is that of Employment Specialists placed within the primary care and mental health services providing intensive support to move people into employment as quickly as possible. Employees and employers are then provided with ongoing support, using the Individual Placement and Support model, for as long as is required to development sustainable employment. Any evaluation of such programmes of more long-term support would need to include a cost-benefit analysis that would incorporate a comparison between providing long-term support to move into employment against the costs of maintaining people on long-term benefits.

Reforms under previous governments focused on reducing the 'flow' onto benefits, without improving assistance to the 'stock' of existing claimants, who make up the vast majority of claimants. Our synthesis of the evidence provides support for the position that existing programmes for long-term ill and disabled people will need to be modified and extended if they are to meet the more complex needs of those further from the labour market.

Finally, it is important to recognise that the emphasis of the recent and planned reform programme in the UK is heavily on the types of individual-oriented interventions reviewed in this paper - concerned with changing the behaviour of potential employees. There is a relative neglect of the environmental orientation which would address real barriers to employment of disabled people and people with long-standing health conditions, including attitudes and practices of employers and agencies, as well as access to work issues. A systematic review of such environmental types of intervention from comparable OECD countries is the subject of a separate paper [56].

## Competing interests

The authors declare that they have no competing interests.

## Authors' contributions

MW was principal investigator and conceived the idea for the study. All authors contributed to aspects of the design and planning of the systematic review. KM designed and conducted the searches. SC, SP and CB were principal reviewers, with assistance from RG, carrying out screening, selection and data extraction. All authors contributed to interpretation of results, writing of the paper and have seen and approved the final manuscript.

## Pre-publication history

The pre-publication history for this paper can be accessed here:

http://www.biomedcentral.com/1471-2458/11/170/prepub

## Supplementary Material

Additional file 1 **Adobe Acrobat file (pdf) providing details of the inclusion and exclusion criteria used to filter the studies located in the electronic and grey literature searches**. Click here for file

Additional file 2 **Adobe Acrobat file (pdf) setting out basic search strategy and details of the electronic databases and UK organisational website searched**. Click here for file

Additional file 3 **Adobe Acrobat file (pdf) providing details of appraisal criteria for both quantitative and qualitative studies used to assess study quality**. Click here for file

Additional file 4 **Adobe Acrobat file (pdf) flowchart detailing numbers of studies located numbers excluded and for what reason**. Click here for file

Additional file 5 **Adobe Acrobat file (pdf) providing implementation details of the return to work initiatives reviewed**. Click here for file

Additional file 6 **Adobe Acrobat file (pdf) table providing details of the studies (authors, dates, intervention types, study design and employment outcomes/other findings) for individual case management and job search assistance interventions**. Click here for file

Additional file 7 **Adobe Acrobat file (pdf) table providing details of the studies (authors, dates, intervention types, study design and employment outcomes/other findings) for financial incentives for disabled people interventions**. Click here for file

Additional file 8 **Adobe Acrobat file (pdf) table providing details of the studies (authors, dates, intervention types, study design and employment outcomes/other findings) for health condition management interventions**. Click here for file
