# Supplementary material for: Assembling the evidence jigsaw: insights from a systematic review of UK studies of individual-focused return to work initiatives for disabled and long-term ill people
Source: BMC Public Health. 2011 Mar 21;11:170. doi: 10.1186/1471-2458-11-170 (PMC3070652; doi:10.1186/1471-2458-11-170)
Supplement: Additional file 1 — Adobe Acrobat file (pdf) providing details of the inclusion and exclusion criteria used to filter the studies located in the electronic and grey literature searches. [file 1471-2458-11-170-S1.PDF]

Additional file 1: Inclusion/Exclusion Criteria

	Included	Excluded
(i) Geographical coverage	UK	All other countries
(ii) Time coverage	Studies published between 2002 and 2007	Pre-2002
(iii) Population of interest	Working age (16-65) population who are chronically ill or disabled.	Pre- or post-working age population, and working age population without a chronic illness or disability
(iv) Studies of interest	1. Empirical evaluations of effectiveness of 'welfare-to-work' programmes on employment.<br>2. Qualitative studies of the views of participants and process evaluations of the implementation of the included interventions.<br>3. Systematic reviews of studies in 1 and 2 above	1. Evaluations of 'welfare-to-work' and 'long-term sick to work' programmes that do not include employment outcomes 2. Studies that do not include empirical data or do not review empirical studies<br>3. Single cross sectional studies
(v) Type of policy intervention	Major 'Welfare-to-work' and 'Return-to-work" programmes and their constituent elements to improve the employment chances of chronically ill or disabled people of working age.	1.Small-scale, local experiments for specific groups of chronically ill or disabled people not part of a national policy initiative;<br>2. "sheltered employment" for severely disabled people that would not constitute employment in the open or competitive labour market.<br>3. "Welfare to work" programmes for people without a chronic illness or disability.<br>4. Programmes to reduce short-term absence rates among the employed population.
(vi) Outcomes	Effectiveness – employment chances and social inclusion<br>Process – influence of contextual factors on implementation<br>Organisation – features of the policy intervention that influence operation	Studies that do not address outcomes in terms of effectiveness, process or organisation.
