# Supplementary material for: Assembling the evidence jigsaw: insights from a systematic review of UK studies of individual-focused return to work initiatives for disabled and long-term ill people
Source: BMC Public Health. 2011 Mar 21;11:170. doi: 10.1186/1471-2458-11-170 (PMC3070652; doi:10.1186/1471-2458-11-170)
Supplement: Additional file 2 — Adobe Acrobat file (pdf) setting out basic search strategy and details of the electronic databases and UK organisational website searched. [file 1471-2458-11-170-S2.PDF]

Additional file 2:

Search Strategy

Identification of literature involved searching a range of electronic and grey literature databases. The search process was undertaken in two stages:
[1] an update of an existing review which looked at welfare-to-work programmes in the UK
[2] a search of relevant organisational and government department web pages.

The strategies were not limited by study design or language. A basic search strategy was designed to retrieve the following facets in combination:
Population - Disability, long term-sickness etc.
AND
Intervention – rehab interventions/programmes
AND
Outcome - Employment/unemployment

Databases searched between 2002-2007:
Medline/Medline In-Process Citations (up to 6.3.07), Embase, HMIC, IBSS, PsycInfo, EconLit, DARE, Cochrane Database of systematic Reviews, Cochrane Central Register of Controlled Trials, Social Science Citation Index, ASSIA, Social Services Abstracts, Sociological Abstracts, Social Care Online, Dissertation Abstracts.
Econpapers was searched separately by a member of the research team.

UK organisational websites searched
Department for Work and Pensions, Department for Education and Skills, Department of Health, Inland Revenue, Social Exclusion Unit, Scottish Executive, Welsh Assembly, the Disability Rights Commission, Centre for Disability Studies, Centre for Analysis of Social Exclusion, Institute for Economic and Social Research, Institute of Employment Studies, Joseph Rowntree Foundation, Social Policy Research Unit, Strathclyde Centre for Disability Research, British Council of Disabled People, Disability Alliance, Royal National Institute for the Blind, Sainsbury Centre for Mental Health, Chartered Institute of Personnel and Development, Social firms UK.

These searches were supplemented through contacting 26 key researchers in the field to request information on unpublished and in-progress research. These key researchers were either authors of relevant studies identified in the searches or key personnel in research and/or employment and disability organisations identified during both the searches and researching the relevant policy literature.

An example Medline search strategy is presented below. The main search strategy was adapted to search the other databases listed above (for details of the full search strategy, please contact Kate Misso kate@systematic-reviews.com).

1. exp Disabled Persons/
2. (disabled or disabilit$).mp.
3. Chronic Disease/
4. (chronic ill$ or chronic absence$ or chronic disease$).mp.
5. chronic sick$.mp.
6. (long standing sick$ or long standing absence$ or long standing disease$).mp.
7. long standing ill$.mp.
8. (longstanding ill$ or longstanding absence$ or longstanding disease$).mp.

9. longstanding sick$.mp.
10. llsi.ti,ab.
11. (long term sick$ or long term absence$ or long term disease$).mp.
12. (longterm sick$ or longterm absence$ or longterm disease$).mp.
13. long term ill$.mp.
14. longterm ill$.mp.
15. (permanent ill$ or permanent absence$ or permanent disease$).mp.
16. permanent sick$.mp.
17. or/1-16
18. Employment/
19. exp Occupations/
20. Work/
21. Unemployment/
22. occupation.mp.
23. occupations.mp.
24. work$.mp.
25. vocation$.mp.
26. (unemploy$ or employ$).mp. or (labour or labor).ti,ab.
27. (job or jobs).mp.
28. (earn$ or paid or paying or payment$).mp.
29. Income/ or "Salaries and Fringe Benefits"/
30. (salary or salari$ or income or wages or waged or wage).mp.
31. or/18-30
32. rehabilitation/ or rehabilitation, vocational/
33. Education, Professional, Retraining/
34. "welfare to work".mp.
35. "back to work".mp.
36. Training Support/
37. training.mp.
38. retraining.mp.
39. re-training.mp.
40. (skill or skills).mp.
41. advice.mp.
42. Counseling/
43. (counselling or counseling).mp.
44. Insurance, Disability/
45. disability benefit$.mp.
46. Insurance, Liability/
47. Social Security/
48. disability pension$.mp.
49. Sick Leave/
50. sick leave.mp.
51. Retirement/
52. mobility allowance$.mp.
53. disabilit$ allowance$.mp.
54. sickness$ benefit$.mp.
55. sickness$ pension$.mp.
56. premature$ retire$.mp.
57. early retire$.mp.
58. (quota or quotas).mp.
59. mobility pension$.mp.

60. invalidity pension$.mp.
61. invalidity allowance$.mp.
62. invalidity benefit$.mp.
63. almp.ti,ab. or active labour market program$.mp.
64. employ$ subsidy.mp.
65. employ$ subsidies.mp.
66. disability living allowance$.mp.
67. attendance allowance$.mp.
68. incapacity benefit$.mp.
69. incapacity allowance$.mp.
70. incapacity pension$.mp.
71. severe disablement allowance$.mp.
72. Employment, Supported/
73. supported work.mp.
74. supported employ$.mp.
75. disabled persons tax allowance$.mp.
76. "new deal for disab$".mp.
77. "new deal for jobseekers with disab$".mp.
78. "access to work program$".mp.
79. "pathways to work".mp.
80. work preparation$.mp.
81. job broker$.mp.
82. permitted work rule$.mp.
83. work focussed interview$.mp.
84. work focused interview$.mp.
85. (workstep or work-step).mp.
86. disability working allowance$.mp.
87. condition management program$.mp.
88. "return to work credit".mp.
89. (work trial or work trials).mp.
90. (employment trial or employment trials).mp.
91. work placement$.mp.
92. "job introduction scheme$".mp.
93. disability discrimination act.mp.
94. "new deal innovative scheme$".mp.
95. work preparation.mp.
96. job preparation.mp.
97. "new deal personal adviser$".mp.
98. "new deal personal advisor$".mp.
99. working tax credit.mp.
100. "travel to work".mp.
101. (jobseeker$ grant$ or job seeker$ grant$).mp.
102. (jobfinder$ grant$ or job finder$ grant$).mp.
103. (jobfinder$ allowance$ or job finder$ allowance$).mp.
104. (jobseeker$ allowance$ or job seeker$ allowance$).mp.
105. (job match or workfare or work fare).mp.
106. "52 week linking rule$".mp.
107. "access to work".mp.
108. (jobmatch or active labor market program$).mp.
109. or/32-108
110. 17 and 31 and 109
