# Supplementary material for: Assembling the evidence jigsaw: insights from a systematic review of UK studies of individual-focused return to work initiatives for disabled and long-term ill people
Source: BMC Public Health. 2011 Mar 21;11:170. doi: 10.1186/1471-2458-11-170 (PMC3070652; doi:10.1186/1471-2458-11-170)
Supplement: Additional file 3 — Adobe Acrobat file (pdf) providing details of appraisal criteria for both quantitative and qualitative studies used to assess study quality. [file 1471-2458-11-170-S3.PDF]

Additional file 3: Critical Appraisal Criteria

Critical Appraisal - Quantitative

1	*Is the study based on a representative sample selected from a relevant population?*
2	*Does the study use an appropriate comparison group?*
3	*Is the baseline response equal or greater than 60% of initial sample?*
4	*Is (a) the follow-up rate in a cohort study equal to or greater than 80% of the baseline response, or (b) is each follow-up survey in a repeat cross-sectional study equal to or greater than 60% of the follow-up sample?*
5	*Are the effects of non-responses and/or drop-outs explored?*
6	*Are the authors conclusions' substantiated by the data in their results*
7	*Are the effects of potentially important confounding factors explored?*
8	*Were all members of the study population (or intervention group) exposed to the intervention?*
9	*Does the study use statistical tests appropriate to the type of data?*

Critical appraisal - qualitative

**1**	Was there a clear statement of the research question and aims?
**2**	Was the research design appropriate to address the aims of the research?
**3**	Does the sample produce the type of knowledge necessary to understand the structures and processes within which the individuals or situations are located?
**4**	Were the data collected in a way that addressed the research issue?
**5**	Are the data analysis methods appropriate to the subject matter?
**6**	Is the description of the findings provided in enough detail and depth to allow interpretation of the meanings and context of what is being studied?
**7**	Are the conclusions justified by the results?
**8**	Have the limitations of the study and their impact on the findings been taken into account?
**9**	Has the relationship between researcher and participants been adequately considered?
