# Supplementary figures and images for: Assembling the evidence jigsaw: insights from a systematic review of UK studies of individual-focused return to work initiatives for disabled and long-term ill people
Source: BMC Public Health. 2011 Mar 21;11:170. doi: 10.1186/1471-2458-11-170 (PMC3070652; doi:10.1186/1471-2458-11-170)

**Additional file 4: Search & Inclusion Flowchart – UK 2002-2007**

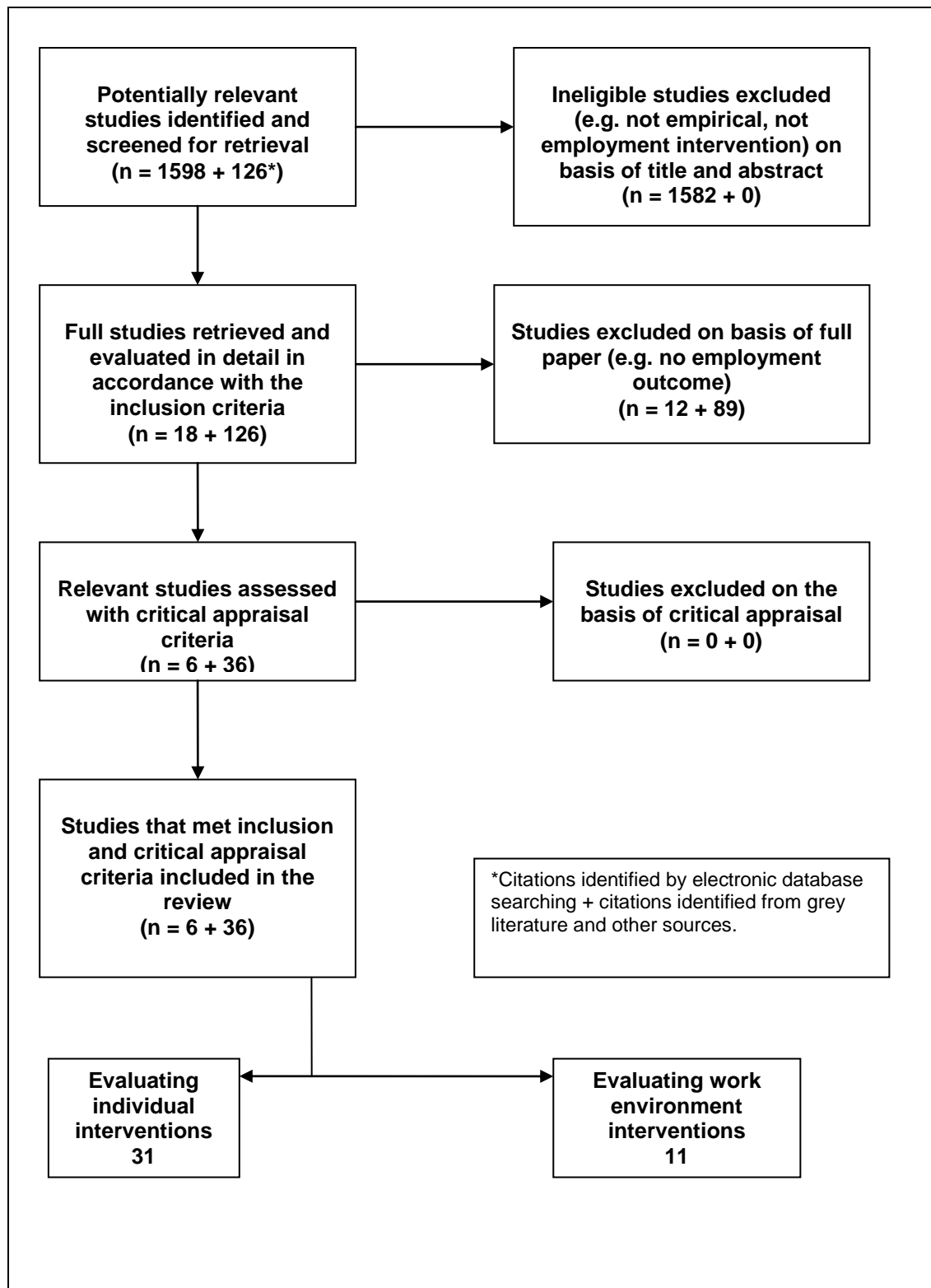

Supplement: Additional file 4 — Adobe Acrobat file (pdf) flowchart detailing numbers of studies located numbers excluded and for what reason. [file 1471-2458-11-170-S4.PDF]
