# Supplementary material for: Assembling the evidence jigsaw: insights from a systematic review of UK studies of individual-focused return to work initiatives for disabled and long-term ill people
Source: BMC Public Health. 2011 Mar 21;11:170. doi: 10.1186/1471-2458-11-170 (PMC3070652; doi:10.1186/1471-2458-11-170)
Supplement: Additional file 5 — Adobe Acrobat file (pdf) providing implementation details of the return to work initiatives reviewed. [file 1471-2458-11-170-S5.PDF]

Additional file 5

Box 1: Intervention implementation details

ONE Advisory Service pilots (1999-2001)
Integrated the Employment Service and Benefits Agency into a single point for unemployed welfare benefit claimants (including those on disability-related benefits) in 12 pilot areas using three models: Basic, Call Centre and Private/Voluntary Sector (PVS). Initially introduced as a voluntary scheme: all new and repeat claimants were assigned a Personal Adviser to process their benefit claim and, through work-focused interviews, review their job readiness, options for work, and barriers to work and provided services such as a better-off calculation and advice about in-work benefits. From April 2000 claimants were required to attend a work-focused meeting as a condition of receiving benefits in the pilot areas.

New Deal for Disabled People (piloted: 1998, national extension: 2001 –?)
Once extended nationally, all incapacity-related benefit claimants were eligible to take part in the New Deal for Disabled People on a voluntary basis. The service was delivered through a network of 65 Job Brokers (charity/voluntary, private and public sector organisations bid for contracts). All incapacity-related benefit claimants voluntarily undertake work-focused interviews to access an individualised package of job search activities, access to appropriate training and other employment advice (including in-work support for those gaining employment).

Pathways to Work (piloted: 2003/2004, national rollout completed: 2008)
Mandatory work-focused interviews for all new and repeat incapacity benefit claimants eight weeks into their claim (voluntary participation open to existing claimants), most then expected to participate in five more interviews at monthly intervals. Non-attendance could result in benefit deductions. Specialist teams of Incapacity Benefit Personal Advisors, Disability Employment Advisors and Work Psychologists provide individualised advice and support to facilitate claimants' return to work, including the Choices package (easier access to existing programmes such as the New Deal, along with new initiatives such as the Return to Work Credit, and the Condition Management Programme).

Return to Work Credit (introduced with Pathways to Work in 2003/04)
A payment of £40/week for up to 52 weeks available to all incapacity benefit claimants returning to new work for 15+ hours /week and earning less than £15,000 p.a.

Permitted Work Rules (originally introduced 2002)
Universal programme which allows all incapacity benefit and income support claimants to work up to 16 hours/week and earn up to £88.50/week for up to 52 weeks (or earn £20/week indefinitely) without losing benefits (income support claimants lose benefits pound for pound on any earnings over £20.

Condition Management Programme (introduced with Pathways to Work)
Programme focused on the three main conditions reported by incapacity benefit claimants – mental ill-health, cardio-vascular and musculoskeletal problems. Commissioned and delivered jointly by Jobcentre Plus and Primary Care Trusts, the Programme uses interventions such as Cognitive Behavioural Therapy and exercise programmes to address anxiety, pain management and lack of confidence, with programme delivery and content varying according to local needs. Claimants referred onto Programme by their Incapacity Benefit Personal Advisor.
