# Supplementary material for: Assembling the evidence jigsaw: insights from a systematic review of UK studies of individual-focused return to work initiatives for disabled and long-term ill people
Source: BMC Public Health. 2011 Mar 21;11:170. doi: 10.1186/1471-2458-11-170 (PMC3070652; doi:10.1186/1471-2458-11-170)
Supplement: Additional file 6 — Adobe Acrobat file (pdf) table providing details of the studies (authors, dates, intervention types, study design and employment outcomes/other findings) for individual case management and job search assistance interventions. [file 1471-2458-11-170-S6.PDF]

Additional file 6:

Table 2: Individual case management and job search assistance interventions

Study	Programme and year of evaluation	Study Details	Employment outcomes/other findings
Green et al (2003)[16]	ONE Advisory Service<br><br>2000/2001	Controlled cohort study (n=4783) new IB claimants in 12 intervention, 12 control areas.	Employment >16 hours per week increased in both intervention (24% to 28%) and control areas (20% to 25%) – no statistically significant difference. Only the Basic model of delivery showed significant (p<0.01) differences in employment rates (16+ hours per week) between the intervention and control areas.
Kirkby and Riley (2003, 2004)[18, 19]	ONE Advisory Service – work-focused Interviews<br><br>1996-2001	Repeat cross-sectional survey, 5% random sample of UK benefit claimants (n=29,451) – 12 intervention and 12 comparison groups	No statistically significant difference in probability of sick/disabled clients leaving IBs between intervention and comparison areas. Early participants in *ONE* left IBs quicker than later participants suggesting a worsening in labour market outcomes when the service should have been improving after any start-up difficulties. No statistically significant differences in outcomes by gender.
Osgood *et al (*2002) [17]	ONE Advisory Service<br><br>2000/2001	103 qualitative interviews with sick/disabled ONE clients	Clients generally positive about the one-stop-shop approach and personalised service, but felt Advisor meeting focused on benefit claims and not work, and often unaware of options for referral to specialist services/support. Advisors deemed job searches inappropriate for sick/disabled clients. Most clients agreed; some felt this was a missed opportunity reflecting Advisors' lack of experience/awareness. Some clients felt Private/Voluntary Sector Advisors lacked expertise. Little evidence of innovative modes of delivery in PVS model.
Kelleher *et al* (2002) [20]	ONE Advisory Service<br><br>2000/2001	Longitudinal, qualitative panel (72 ONE staff), observations of 175 client-staff meetings in three pilot sites and staff focus groups	Staff positive, but felt meetings too focused on benefit claims and limited ability to provide individualised approach for sick/disabled clients. Main barriers to delivery: inadequate staffing levels meant extended client waiting times which limited time build rapport with clients and for job search activities; job placement targets; excessive bureaucracy. Private and voluntary sector managers felt ability to innovate limited by contractual arrangements and time and resource issues.

Orr *et al* (2007) [21]	New Deal for Disabled People<br><br>2001-2004	Controlled cohort NDDP eligible population registrants (n=522,596) and non-registrants (n=44,049)	24 months after NDDP registration employment rates increased 11% for existing IB recipients and 7% for new claimants (p<0.05). Benefit recipiency decreased 16% for existing claimants and 13% for new claimants (p<0.001). Employment effects stronger for those claiming IB for at least 3 years, those furthest from the labour market and in areas of high economic inactivity. No significant differences by *Job Broker* type.
Kazimirski *et a*l (2005) [24] ; Adelman *et al* (2004) [22]; Ashworth *et al* (2003) [23]	New Deal For Disabled People<br><br>Job Brokers (JBs)<br><br>2002 - 2004	Uncontrolled, cohort survey (n=4082) NDDP registrants.	12 months after intervention 47% of registrants in paid work (19% under *Permitted Work*). Women (OR 1.238, p<0.01), over-50s (OR 1.392, p<0.05), those who do not have problems with basic skills (OR 1.397, p<0.01), those with health conditions not very limiting (OR 1.988, p<0.05) more likely to be employed. Ethnic minorities (OR 0.729, p<0.05), those without musculoskeletal (OR 0.496, p<0.001) or mental health conditions (OR 0.648, p<0.05) less likely to be employed. Registrants in London (OR 0.520, p<0.001), the North West (OR 0.660, p<0.001), and the West Midlands (OR 0.472, p<0.001) less likely to be employed. 72% of employed participants felt JBs not reason for gaining employment.
Woodward *et al* (2003) [32]; Pires *et al* (2006) [31]	New Deal for Disabled People<br><br>Job Brokers (JBs)	Repeat cross-sectional telephone survey of NDDP eligible population(n= 3,452); 30 in-depth interviews with 'knowledgeable' non-registrants	Low awareness of NDDP/JBs, under 50% of eligible population at last survey wave. 5% respondents' reported registration, number of registrants on NDDP database much lower. Non-registration largely due to ill-health and fears of losing benefits.
Aston *et al* (2003, 2005) [27, 28]	New Deal for Disabled People<br><br>Job Brokers<br><br>2002-2004	Two waves of in-depth interviews (wave 1, n80, wave 2, n50) with employers participating in NDDP	Low awareness, less than ¼ employers reported being involved with NDDP or JBs. Generally providing NDDP participants with low-level clerical, customer service or call centre work. By building close working relationships with potential employers, JBs could change attitudes toward recruiting people with disabilities.

Lewis et al (2005) [26]; Corden et al (2003) [25]	New Deal For Disabled People<br><br>Job Brokers<br><br>2002 - 2004	Qualitative longitudinal study – two waves: interviews with 135 JB clients, 18 JB managers, 23 Disability Employment Advisors, 32 staff focus groups	Claimants requesting and gaining high levels of support felt JBs had positive impact on their move toward/into work. Other factors (e.g. clients' health, access to transport, caring role), limited employment impacts. Employment gained usually part-time (<35 hours per week), un- or semi-skilled, usually lower level than before being on benefits, due to health conditions and/or to remain within the *Permitted Work Rules*.
Heenan (2003) [30]	New Deal for Disabled People<br><br>Personal Advisors<br>(no date)	2 focus groups – 28 users of charitable sector pilot Personal Advisor scheme	Delivery organisation key factor in IB claimants' decision to participate – preference for charitable/voluntary sector delivery due to disillusion with and lack of trust in state sector and fear of benefit withdrawal (small sample size).
Davis *et al* (2006) [29]	New Deal For Disabled People<br><br>Job Brokers<br><br>2005	21 in-depth interviews with Jobcentre Plus (JcP) and Job Broker (JB) Managers, 5 JcP staff focus groups; performance assessment: conversion rates of registrations to job entries 2001-2005.	Performance targets, relationship between JB and JcP, JB size, location and premises, quality and commitment of staff, knowledge of local labour market all created erratic JB performance levels. Performance targets may have driven selection of the most job ready clients to maintain performance figures.
Adam *et al* (2006) [33]	Pathways to Work Pilots<br><br>2003-2005	Controlled cohort (n=8,035) claimants making initial inquiry about IB in seven *Pathways* pilot areas.	10½ months after initial inquiry, intervention group employment probability increased (9.4%, $p<0.001$), monthly earnings increased (+£71.73, $p<0.001$), reduced probability of claiming IBs (-8.2%, $p<0.001$) and of reporting a limiting health problem (-2.9%, $p<0.05$). No statistically significant employment differences by sex or age.
Bewley *et al* (2007) [34]	Pathway to Work Pilots<br><br>2003-2006	Controlled cohort (n=5,784) claimants making initial inquiry about IB in seven *Pathways* pilot areas	18 months after initial inquiry, intervention group employment probability increased (7.4%, $p=0.09$), no significant effect on earnings, probability of claiming IBs or probability of reporting limiting health problem. Employment effect stronger for women (13% $p<0.05$) and those with dependent children (17.6%, $p<0.05$).

Corden *et al* (2005) [42]; Corden & Nice (2006a, 2006b) [40, 41]	Pathways to Work Pilots<br><br>Work-focused Interview (WFI)<br><br>Job Brokers (JB)<br><br>2004-2006	Longitudinal qualitative panel study with 3 cohorts IB recipients (n=105) in seven pilot areas.	Claimants closest to work viewed WFI as valuable help and support, others, (especially older respondents), found Programme of no use. A third group viewed scheme as no more useful than previous interventions. Similar views of JBs and no evidence of employment gained through JBs. Most claimants felt if own health improved then more likely to move toward work – *Pathways* had little impact on this.
Dickens *et al* (2004a) [37]	Pathways to Work Pilots<br><br>Work-focused Interview (WFI)	6 focus groups (3 with 13 new IB customers, 3 with 18 Incapacity Benefit Personal Advisors) in 3 initial pilot areas	Early implementation findings. Advisors felt WFIs provide consistent support to IB customers – though need more time for those furthest from the labour market. Less customer resistance to WFIs than expected. Some concerned mandatory WFIs and targets might cause tensions. Claimants were positive about WFIs where provision fitted their needs - emphasised Advisors need to listen and understand their health condition.
Dickens *et al* (2004b); Knight *et al* (2005) [36, 39]	Pathways to Work Pilots<br><br>Incapacity Benefits Personal Advisors (IBPAs)<br><br>Job Brokers<br><br>2004 - 2005	56 in-depth interviews with Advisors and work psychologists 10 Advisor focus groups over two waves in seven pilot areas	Advisors skills and approach to their work and ability to establish good relationship key to changing claimants' attitudes and moving them towards work. Could be undermined by job outcome targets and inherent tension between their role as 'enforcers' and 'enablers'. Advisors felt JBs could only help those claimants closest to labour market and so selected those claimants for referral.

Barnes & Hudson (2006a) [35]	Pathways to Work – extension to existing customers	Focus groups (13 claimants) 5 in-depth claimant interviews, 19 telephone interviews with Advisors	Claimants mostly positive about WFIs and viewed Advisors as keen and helpful. Many felt *Pathways* could not overcome main barriers to work (weak local labour markets, attitudes of employers). Those too ill felt they had nothing to gain. Advisors viewed existing claimants distant from labour market and worse (often mental) health conditions, so required more long-term engagement, but managers unaware of efforts needed to achieve progress these clients and inappropriateness of job outcome targets.
Dixon *et al* (2007) [38]	Pathways to Work – extension to existing customers	Observation of 17 WFIs, follow-up interviews with matched Advisors (13) and IB claimants (17)	Too early to report job outcomes. Advisors and claimants identified building trust as central to positive outcomes. Most Advisors felt early work outcomes could not be expected from existing claimants with complex and multiple barriers to work. Some unclear whether moving toward work is a justifiable outcome under *Pathways*.
	2006		
