# Supplementary material for: Assembling the evidence jigsaw: insights from a systematic review of UK studies of individual-focused return to work initiatives for disabled and long-term ill people
Source: BMC Public Health. 2011 Mar 21;11:170. doi: 10.1186/1471-2458-11-170 (PMC3070652; doi:10.1186/1471-2458-11-170)
Supplement: Additional file 7 — Adobe Acrobat file (pdf) table providing details of the studies (authors, dates, intervention types, study design and employment outcomes/other findings) for financial incentives for disabled people interventions. [file 1471-2458-11-170-S7.PDF]

Additional file 7

Table 3: Financial incentives for disabled people

Study	Programme and year of evaluation	Study Details	Employment Outcomes
Corden *et al* (2005) [42]; Corden & Nice (2006a, 2006b) [40, 41]	Return to Work Credit (RTWC)<br><br>2004-2006	Longitudinal qualitative panel study with 3 cohorts IB recipients (n=105) in seven *Pathways* pilot areas.	Limited take up of RTWC; primarily women returning to low skill part-time work. Limited incentive effect as need to be in work. Extra income can be eroded by loss of other benefits (e.g. housing) debt repayments or recovery of unpaid income tax.
Corden & Nice (2006c) [43]	Return to Work Credit	35 in-depth interviews with RTWC recipients across 7 pilot areas.	RTWC may support lasting transitions to work from IBs through earnings supplement effect and helping clear debts. No clear evidence of incentive effect - amount too low in comparison to overall household income. Those keen to move into work often do so without knowing about RTWC.
Dickens *et al* (2004b); Knight *et al* (2005) [36, 39]	Return to Work Credit<br><br>2004 - 2005	56 in-depth interviews with Advisors and work psychologists 10 Advisor focus groups over two waves in seven pilot areas	Advisors positive about RWTC – viewed as incentive and support for claimants able to work part-time. Uptake was highest amongst voluntary customers closest to labour market. Some Advisors concerned low paid work unsustainable after RWTC ended.
Dewson *et a*l (2004, 2005) [45, 46]	Permitted Work Rules (PWR)<br><br>2003/2004/2005	Uncontrolled cohort of IB recipients (n=1435) undertaking PWR in 2002 + longitudinal in-depth interviews with 59 PWR participants	2005 – 25% respondents in paid work not claiming IB, 33% in paid work and claiming IB under PWR. No statistically significant association between being in work and age or gender. Longer IB claim duration, less likelihood of being in work either independently or under Permitted Work. 47% with a working partner in paid work not claiming IBs, compared to 37% of single people and 15% with economically inactive partner.
