# Supplementary material for: Assembling the evidence jigsaw: insights from a systematic review of UK studies of individual-focused return to work initiatives for disabled and long-term ill people
Source: BMC Public Health. 2011 Mar 21;11:170. doi: 10.1186/1471-2458-11-170 (PMC3070652; doi:10.1186/1471-2458-11-170)
Supplement: Additional file 8 — Adobe Acrobat file (pdf) table providing details of the studies (authors, dates, intervention types, study design and employment outcomes/other findings) for health condition management interventions. [file 1471-2458-11-170-S8.PDF]

Additional file 8

Table 4: Health condition management

Study	Programme and year of evaluation	Study Details	Employment Outcomes
Barnes & Hudson (2006b) [47]	Condition Management Programme<br><br>2005	37 in-depth interviews with CMP staff in seven *Pathways* pilot areas.	Practitioners reported full spectrum of progress from gaining employment to not engaging –successful outcomes result from combined CMP and Jobcentre Plus interventions – those not progressing needed more specialist help. Reports that practitioners selected only those claimants they thought would benefit.
Corden *et al* (2005) [42]; Corden & Nice (2006a, 2006b) [40, 41]	Condition Management Programme<br><br>2004-2006	Longitudinal qualitative panel study with 3 cohorts IB recipients (n=105) in seven pilot areas.	CMP viewed positively but relatively little take up across three cohorts and lack of understanding of the purpose of CMP. Few who returned to work credited CMP. Specific condition CMPs more effective than generic schemes.
Dickens *et al* (2004b) [36]; Knight *et al* (2005) [39]	Condition Management Programme<br><br>2004 - 2005	56 in-depth interviews with Advisors and work psychologists , 10 Advisor focus groups over two waves in seven pilot areas	Referrals to CMP varied, according to Advisor understanding of CMP improved at 2nd wave interviews. Advisors remained unclear which claimants suitable for CMP. Advisors felt CMP key to moving forward claimants not yet ready to consider work, but prepared to overcome initial barriers.
Dixon *et al* (2007) [38]	Condition Management Programme<br><br>2006	Observation of 17 WFIs, in-depth follow-up interviews with matched Advisors (13) and IB claimants (17)	Advisors viewed CMP as most appropriate option for existing customers as managing their health condition was viewed as first step toward employment. Still evidence of a lack of clarity in referrals due to poor knowledge of health conditions (esp. mental) and how CMP operates. Customers largely positive, but did not feel closer to employment.
